# Early structural changes during spontaneous closure of idiopathic full-thickness macular hole determined by optical coherence tomography: a case report

**DOI:** 10.1186/1756-0500-6-396

**Published:** 2013-10-02

**Authors:** Akiko Okubo, Kazuhiko Unoki, Keita Yamakiri, Munefumi Sameshima, Taiji Sakamoto

**Affiliations:** 1 Unoki Eye Clinic, Harara 1-7-15, Kagoshima 890-0026, Japan; 2 Kagoshima University Graduate School of Medical and Dental Sciences, Kagoshima, Japan

**Keywords:** Idiopathic full thickness macular hole, Spontaneous resolution of macular hole, Optical coherence tomography, Müller cell, External limiting membrane

## Abstract

**Background:**

Spontaneous closure of an idiopathic full-thickness macular hole has been reported to occasionally occur. However, the cells involved in plugging the macular hole have not been determined conclusively. We aimed to report the early structural changes that occur during a spontaneous closure of an idiopathic full-thickness macular hole determined by spectral-domain optical coherence tomography.

**Case presentation:**

A 71-year-old Japanese man with an idiopathic full-thickness macular hole and subclinical posterior vitreous detachment in the left eye was followed. Three weeks after the identification of the macular hole, optical coherence tomography showed tissue that protruded from the interior wall of the macular hole at the level of the external limiting membrane toward the center of the macular hole. Five months after the first examination, he returned with improvements of his visual symptoms, and the macular hole was closed by a thin retinal tissue which included the restored external limiting membrane that bridged across the macular hole. However, the inner segment/outer segment junction line was not intact and the fovea was detached. Two months later, optical coherence tomography showed an almost normal foveal configuration with an essentially restored inner segment/outer segment junction line and foveal reattachment.

**Conclusion:**

Our results suggest that Müller cells proliferate and/or extend at the level of the end of the external limiting membrane to form a tissue bridge across the macular hole associated with the external limiting membrane restoration first of all. This leads to the adhesion of other retinal layers and resolution of the foveal detachment.

## Background

A spontaneous closure of an idiopathic full-thickness macular hole (MH) occasionally occurs [1,2]. Examining the structural changes of the retina that lead to the closure, especially what types of cells are involved in plugging the MH, would help in the understanding of the mechanisms and the cells involved in the spontaneous closures. Although no histopathologic studies of spontaneously and completely closed idiopathic full-thickness MHs have been published, clinicopathological reports regarding MHs closed by vitrectomy suggest that Müller cells and astrocytes would be the most likely candidates in the repair of a MH [3,4]. Several investigators have followed the processes involved in the closure of a MH and restoration of the macular architecture by optical coherence tomography (OCT) [5-7]. However, the cells involved in the early structural changes that form the bridge over a MH have still not been determined conclusively.

We document the sequence of structural changes, including the early changes, that occur during a spontaneous closure of idiopathic full-thickness MH by spectral-domain OCT (SD-OCT). Our findings suggest that Müller cells play the initial role in the closure and restoration of the outer retina.

## Case presentation

**Figure 1:**
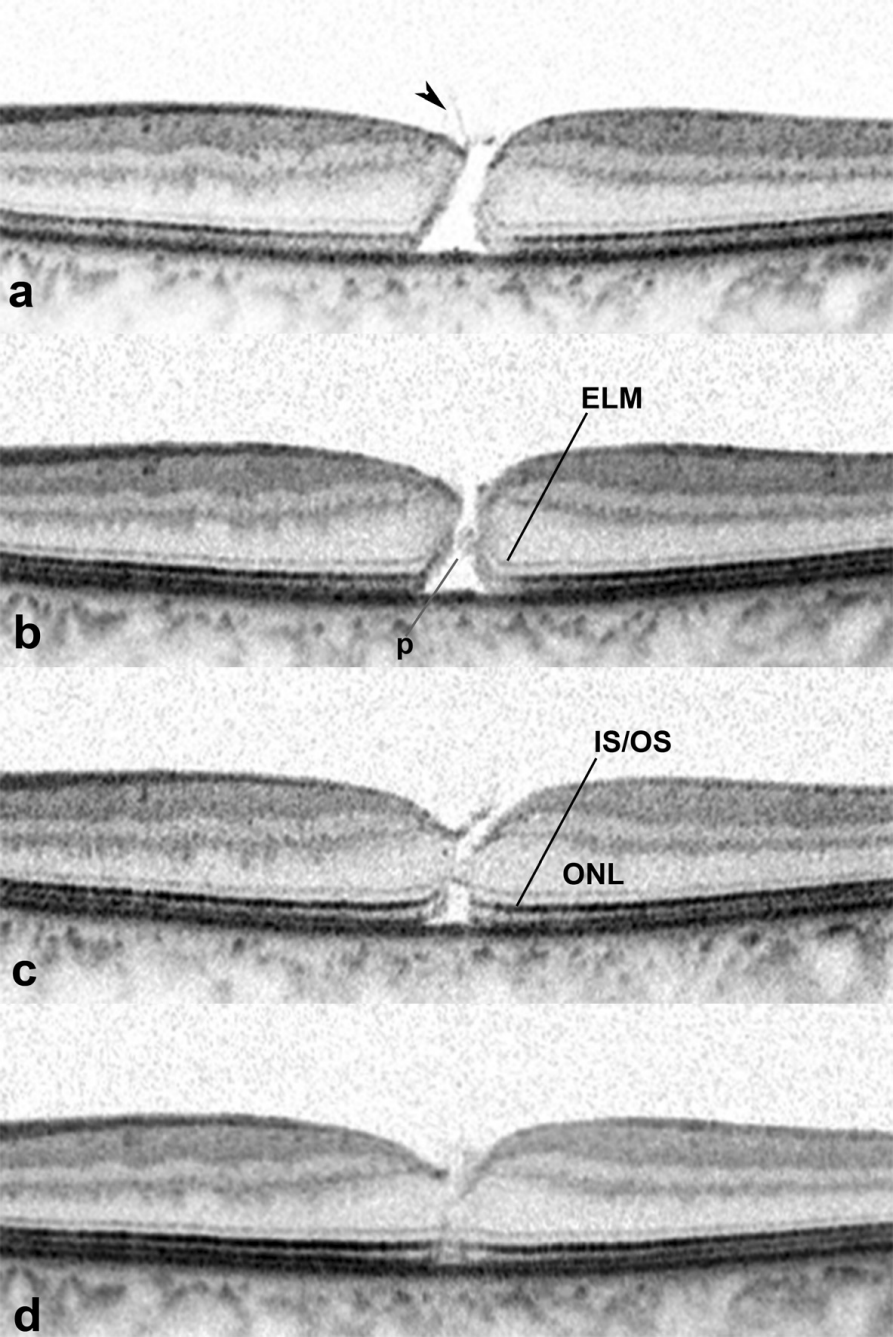
**Spontaneous closure of a macular hole (MH) in the left eye of a 71-year-old man with an idiopathic full-thickness macular hole and subclinical posterior vitreous detachment. a**: Initial examination. Spectral-domain optical coherence tomographic (SD-OCT) scan passing through the fovea horizontally showing a full-thickness MH. The diameter of the MH at the level of the retinal pigment epithelium was 396 μm. A membranous tissue (arrowhead), probably the internal limiting membrane and/or part of the vitreous cortex is seen adhered to the retinal surface at the MH margin. Neither a pseudo-operculum nor intraretinal cysts is seen. **b**: Three weeks later, SD-OCT showed tissue (p) protruding from the interior wall of the MH. The tissue extends from the end of the elevated and disrupted external limiting membrane (ELM) toward the opposite side of the MH. **c**: SD-OCT at 5 months after the initial visit showing a MH closed by thin retinal tissue that extends from the outer portion of the outer nuclear layer (ONL) including the ELM across the MH. An inner segment/outer segment (IS/OS) junction defect and foveal detachment are present. **d**: Seven months after the initial visit, the IS/OS junction defect had essentially recovered. The foveal detachment had recovered.

A 71-year-old man who was taking medications for hypertension and Parkinson’s disease presented with a 5-week history of metamorphopsia of the left eye. His best-corrected visual acuity was 20/40 in the right eye and 20/80 in the left eye. He did not report any trauma before the metamorphopsia. Fundus examination of his left eye showed a small MH without a Weiss ring. SD-OCT (HRA + OCT: Heidelberg Engineering, Germany) confirmed the full-thickness MH (Figure 1a) and a subclinical posterior vitreous detachment. A membranous tissue, probably the internal limiting membrane (ILM) and/or residual vitreous cortex, was seen adherent to the retinal surface at the MH margin.

Three weeks later, SD-OCT showed tissue that protruded, toward the opposite side of the MH, from the interior wall of the MH at the level of the end of the elevated and disrupted external limiting membrane (ELM) (Figure 1b). Five months after the first visit, he returned with improvements of his visual symptoms and a visual acuity of 20/40. The MH was closed by a bridge of thin retinal tissue that extended from the outer part of the outer nuclear layer across the MH. The ELM was restored, appearing along the outer surface of the bridging tissue, but the inner segment/outer segment (IS/OS) junction line was still not complete. A small foveal detachment was present (Figure 1c). Two months later, the foveal detachment and the IS/OS junction defect had essentially recovered (Figure 1d).

## Conclusions

We were able to follow the sequence of structural changes that occurred during a spontaneous closure of an idiopathic full-thickness MH by SD-OCT. Recent OCT studies have shown the morphological changes including the bridging retinal tissue over a MH and recovery of the outer retina [5-7]. In our case, the retinal tissue protruded from the interior wall of the MH at the level of the end of the ELM. No description of such early morphological changes has been reported.

The positional relation between the protruding tissue and the ELM suggests several possibilities for the tissues involved. The photoreceptors can be excluded because they do not proliferate. We cannot exclude the astrocytes which are present in the inner retina and are known to be mobilized and participate in the formation of retinal glial scars in pathological retinas. However, their possible relation to the ELM cannot be explained. We postulate that the protruding tissue was made up of Müller cells which proliferated and/or extended centripetally. Müller cells are giant cells which occupy the full thickness of the retina from the ILM to the ELM, where they form the zonulae adhaerentes between photoreceptors and Müller cells, or between Müller cells [8].

We observed retinal tissue bridging across the MH accompanied with the recovery of the ELM. This was followed by restoration of the IS/OS junction line and foveal reattachment. These findings are consistent with earlier reports [5-7]. The time-course of each event seen during the closure may be important, although its significance has not been fully discussed. Our hypothesis that the protruding tissue represents extensions of Müller cells would explain the recovery of the ELM associated with the bridging of retinal tissue. Because the ELM is formed by adherence junctions between Müller cells and photoreceptors, or between Müller cells as mentioned above, the ELM should be seen in the OCT images after Müller cells reach the photoreceptors. A centripetally tractive action produced by the extension and/or proliferation of the Müller cells toward the center of the MH could lead to adhesion of other disrupted retinal layers including the IS/OS junction. A bridging by Müller cells might facilitate the recovery of the foveal detachment by pressing the elevated tissue to the retinal pigment epithelium. This would lead to photoreceptor activation and IS/OS junction restoration. We associate these events with the fact that photoreceptor maturation follows ELM formation during normal retinal development [9].

In conclusion, our results suggest that the bridging and ELM restoration by Müller cells during the early period would be a key event for promoting complete closure of a MH.

## Consent

Written informed consent was obtained from the patient for publication of this case report and accompanying images. A copy of the written consent is available for review by the Editor-in-Chief of this journal.

## Abbreviations

MH: Macular hole; SD-OCT: Spectral-domain optical coherence tomography; ELM: External limiting membrane; IS/OS: Inner segment/outer segment; ILM: Internal limiting membrane.

## Competing interests

The authors declare that they have no competing interests.

## Authors’ contributions

AO was the main physician responsible for the patient and a major contributor in writing the manuscript. KY and KU performed retinal examination and OCT evaluation. MS and TS participated and helped in reviewing literature sources for this manuscript and writing this manuscript. All authors read and approved the final manuscript.
